# Internal validation of self-reported case numbers in hospital quality reports: preparing secondary data for health services research

**DOI:** 10.1186/s12874-024-02429-6

**Published:** 2024-12-31

**Authors:** Limei Ji, Max Geraedts, Werner de Cruppé

**Affiliations:** Institute for Health Services Research and Clinical Epidemiology, Philipps-Universität Marburg, Karl-von-Frisch-Strasse 4, Marburg, 35043 Germany

**Keywords:** German hospital quality report, Minimum case volume regulations, Internal data validation, Cross-field validation, Error source analysis, Secondary data

## Abstract

**Background:**

Health services research often relies on secondary data, necessitating quality checks for completeness, validity, and potential errors before use. Various methods address implausible data, including data elimination, statistical estimation, or value substitution from the same or another dataset. This study presents an internal validation process of a secondary dataset used to investigate hospital compliance with minimum caseload requirements (MCR) in Germany. The secondary data source validated is the German Hospital Quality Reports (GHQR), an official dataset containing structured self-reported data from all hospitals in Germany.

**Methods:**

This study conducted an internal cross-field validation of MCR-related data in GHQR from 2016 to 2021. The validation process checked the validity of reported MCR caseloads, including data availability and consistency, by comparing the stated MCR caseload with further variables in the GHQR. Subsequently, implausible MCR caseload values were corrected using the most plausible values given in the same GHQR. The study also analysed the error sources and used reimbursement-related Diagnosis Related Groups Statistic data to assess the validation outcomes.

**Results:**

The analysis focused on four MCR procedures. 11.8–27.7% of the total MCR caseload values in the GHQR appeared ambiguous, and 7.9–23.7% were corrected. The correction added 0.7–3.7% of cases not previously stated as MCR caseloads and added 1.5–26.1% of hospital sites as MCR performing hospitals not previously stated in the GHQR. The main error source was this non-reporting of MCR caseloads, especially by hospitals with low case numbers. The basic plausibility control implemented by the Federal Joint Committee since 2018 has improved the MCR-related data quality over time.

**Conclusions:**

This study employed a comprehensive approach to dataset internal validation that encompassed: (1) hospital association level data, (2) hospital site level data and (3) medical department level data, (4) report data spanning six years, and (5) logical plausibility checks. To ensure data completeness, we selected the most plausible values without eliminating incomplete or implausible data. For future practice, we recommend a validation process when using GHQR as a data source for MCR-related research. Additionally, an adapted plausibility control could help to improve the quality of MCR documentation.

**Supplementary Information:**

The online version contains supplementary material available at 10.1186/s12874-024-02429-6.

## Background

Health services research often relies on secondary data as the basis for its analyses. Guidelines for research using secondary data recommend describing the data source and report on its quality [1, 2] in terms of data availability, consistency, and plausibility, as well as dataset completeness, validity, and possible bias [3]. As validation methods, logical control is used to determine unreasonable values [4], case-basis identification and proportional estimations are used to check for data completeness [4], and descriptive analysis is used to recognise error patterns [5]. A common practice for dealing with implausible values in secondary data is to remove them [4]. However, this is not suitable for studies that require data completeness. Notably, finding the correct value of an error is difficult for the internal validation of datasets, if not impossible. The possibility for correcting data depends on data richness and multifacetedness, or, more precisely, on available redundant information of further variables in the dataset that allows data cross-field comparison. In their absence, statistical methods are often used to deal with missing values, through data imputation or maximum likelihood methods [6].

Minimum caseload requirements (MCR) serve as a quality instrument in health policy. Compliance with MCR allows hospitals to continue performing specific procedures in the following year. To evaluate MCR, secondary data is usually used. The rationale behind MCR is based on the volume outcome relationship observed in several medical procedures. This relationship indicates that hospitals performing a medical procedure more frequently, that is, with a higher caseload, tend to achieve higher quality outcomes [7–11]. In Germany, MCR were introduced for all hospitals in 2004 by the Federal Joint Committee, the highest decision-making body of the joint self-government in the German healthcare system [12]. As of 2023, the MCR set minimum caseloads for nine medical interventions defined by Operation and Procedure Classification Codes (OPS codes, from the German term Operationen- und Prozedurenschlüssel) [12] which the performing hospital must meet annually at the hospital site level.

Hospitals are required to report minimum caseloads in their compulsory and structured annual quality report, called the German Hospital Quality Reports (GHQR) [13]. Hence, the publicly available GHQR are the official data source on hospitals’ MCR compliance. Earlier studies evaluating the MCR compliance of hospitals in Germany appraised the MCR caseload data quality in GHQR with an internal validation approach and stated incomplete and implausible data in the GHQR [14, 15] particularly hospitals with small MCR caseloads were underreported [16, 17]. However, the existing literature lacks a comprehensive and detailed quantitative assessment of this constrained validity. To enhance data quality, the Federal Joint Committee introduced a basic plausibility control for reported MCR caseloads in 2018. Hospitals reporting MCR-relevant OPS in their GHQR data set are expected to specify MCR caseloads, if not they receive a plausibility warning to revise their data [13]. The effect of this plausibility control on data quality for MCR caseloads has not been evaluated yet.

An independent external data source can be used to validate the research data. The official Diagnosis-Related Groups (DRG) Statistic [18] serves as a secondary data source for hospital caseloads, which represents all remunerated inpatient cases in Germany. However, this dataset does not provide data at the hospital site level as the GHQR data, and data protection regulations impede hospital caseload data transparency since hospitals are pseudonymised. Collecting primary data for validation is also not feasible due to its highly time- and resource-consuming nature. Therefore, an internal validation of the MCR caseload data quality in the GHQR is the adequate methodological approach, given that hospitals provide further variables, such as OPS data in the GHQR data allowing cross-field data comparisons.

The aim of this study was to validate MCR caseloads in the GHQR by applying an internal data validation approach, including a data quality detection process and a data correction step as recommended by the relevant guidelines for secondary data analysis [3]. In the final step, the original and validated MCR caseload data were assessed at the federal level through an aggregated caseload comparison with the external secondary data source of DRG Statistic.

## Methods

We leveraged the data richness and related possibility of cross-field comparison within the GHQR dataset to compare the reported values across sections within GHQR, assess the data availability and consistency (detection process), and correct implausible data by choosing more plausible values among several equivalent values reported in other sections (data correction process). The validation outcome enabled us to evaluate the data completeness and informed us of possible error sources in the MCR caseload values. Finally, we compared the internally validated data with the external secondary data source of the DRG at the federal level.

This study analysed four MCR procedures that have been in force with constant MCR thresholds since 2006: complex interventions on (1) the oesophagus and (2) pancreas (MCR threshold = 10 cases per year and hospital site, respectively), (3) stem cell transplantation (25 cases per year and hospital site), and (4) total knee replacement (50 cases per year and hospital site) for the period 2016–2021. Henceforth, these MCR procedures are referred to as ‘oesophagus’, ‘pancreas’, ‘stem cells’, and ‘knee’, respectively.

### Data source 1: GHQR data

GHQR data are provided by hospitals on a self-reporting basis as required by law, with reports being submitted every two years since 2004 and annually since 2012. The Federal Joint Committee is responsible for the publication and dissemination of the data to the public. The reports are presented at the hospital site level. From 2010 to 2019, additional association reports were required for hospitals with more than one site, which basically string together all the individual site reports of a hospital association. The GHQR are publicly accessible in PDF format on the website of the Federal Joint Committee and on the hospitals’ websites. They are also available in .xml format as annual datasets from the Federal Joint Committee upon request.

GHQR contain three parts. Part A gives the general administrative hospital (site) information, including hospital ID, name, address, medical departments, number of beds, number of medical staff in full time equivalents, and inpatient and outpatient case number. Part B presents information at medical department level, including all performed OPS and International Classification of Diseases (ICD) codes with frequencies. Part C contains information on quality assurance, including data on MCR and quality indicators (QIs). QIs are calculated by a third-party organisation in charge of the external quality assurance for hospitals in Germany [13].

**Fig. 1:**
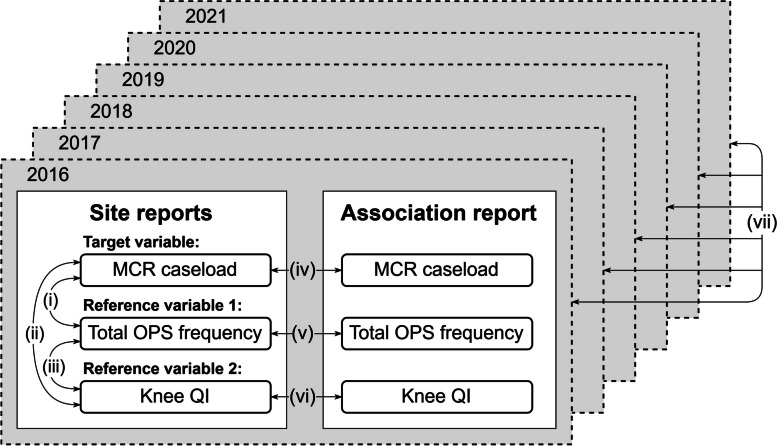
Internal cross-field comparable variables of the German Hospital Quality Reports (GHQR) for data quality detection and data correction. Arrows (i), (ii), (iii): The three key variables in the site reports (MCR caseload, total OPS frequency, and knee QI case number) share similar target values, and their comparison constitutes the internal cross-field validation. Arrows (iv), (v), (vi): The hospital association report, as a summary of the site reports, adds another dimension of internal validation through comparison. Arrow (vii): The chronological caseload values for each hospital site during the study period (2016–2021) serve as an additional validation reference

**Fig. 2:**
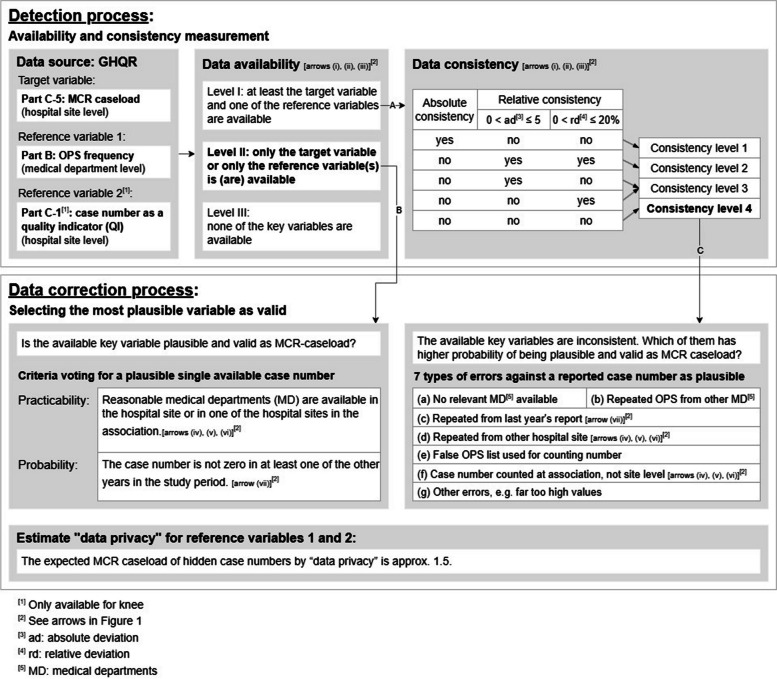
Work modules of the internal validation pertaining to detection and data correction process

We accounted for the following *MCR-related key variables* (‘site reports’ in Fig. 1 and ‘data source: GHQR’ in Fig. 2): (1) MCR caseload in Part C-5 as *the target variable* (hospital site-level variable) to be validated, (2) frequency of performed OPS in Part B as *reference variable 1* (medical department-level variable, aggregated into hospital site-level variable), and (3) case number of a QI ‘indication for elective knee endoprosthesis – primary implantation’ (ID 54020) in Part C-1, as *reference variable 2* (hospital site-level variable); no QI are available for the other three MCR procedures. These three key variables (MCR caseload, OPS frequency, and QI case number) share a similar target value, and the comparison of the three key variables constitutes the internal cross-field validation (arrows (i), (ii), (iii) in Fig. 1).

We defined a tolerance range for the data value comparison for two reasons. First, it is not uncommon for more than one OPS to be performed in one operation. No guideline has clarified how these OPS should be counted as caseload. Second, department-specific OPS numbers cannot show small case numbers (1 ≤ case number ≤ 3), per data privacy requirements. Notably, MCR caseloads are reported without this restriction [12]. The tolerance range is described in the section ‘Detection process’.

In Germany, hospitals are distinguished by their institution identification code (IIC). A hospital with more than one site is referred to as a hospital association. Hospital sites in the same association share the same IIC and are distinguished by an additional site code. We used the association report, as the summary of the site reports, as a reference for the data validation (arrows (iv), (v), (vi) in Fig. 1). In exceptional cases, a hospital/hospital association can have more than one IIC. This had no bearing on the use of GHQR data, only on the IIC-pseudonymised DRG data (see section ‘Data source 2: DRG data’). The chronological caseload values of a hospital site in the study period from 2016 to 2021 served as a further reference source (arrow (vii) in Fig. 1). The linking methodology for creating the longitudinal GHQR database is described elsewhere [19].

The GHQR data calculations were performed using Python 3.10.

### Detection process

We performed the detection process on the data availability and consistency of the *target variable*—MCR caseload—by comparing it with *reference variable 1* and, additionally only for the knee, with *reference variable 2*. The comparisons are shown by arrows (i), (ii), and (iii) in Fig. 1. The detection for the knee involved all three arrows, whereas that for the other three MCR procedures is shown only by arrow (i). In this study, data not available refers to key variables with a value of zero. This is equivalent to not reporting on the key variable. The cross-field comparisons yielded *three availability levels* (see also ‘Data availability’ in Fig. 2), resulting in different data correction processes:

- Availability level I: The target variable is available. At least one of the reference variables is available. In this case, *the consistency of the available key variables* is to be measured.
- Availability level II: Only the target variable or only one or two of the reference variables is available. Thus, the *plausibility of the available key variable(s)* will be checked (see section ‘Data correction process’). This availability level often indicates possible under-reporting.
- Availability level III: None of the key variables are available. The hospital site represented by the dataset is probably *irrelevant to the current MCR procedure*.

For data with availability level I, we categorised the *consistency between the target and reference variables* using the tolerance range with the threshold combinations of (1) no deviation, (2) absolute deviation of five cases, and (3) relative deviation of 20% of cases, resulting in four *consistency levels* (see arrow A and ‘Data consistency’ in Fig. 2):

- Consistency level 1: The target and reference variables are consistent and show no deviation.
- Consistency level 2: The deviation between the target variable and the only available reference variable—or, if both reference variables are available, the smaller deviation between the target variable and each of the two reference variables—is ≤ 5 cases and ≤ 20% at the same time.
- Consistency level 3: The aforementioned deviation is ≤ 5 cases and > 20% or ≤ 20% and > 5 cases.
- Consistency level 4: The aforementioned deviation is > 5 and > 20%.

We considered consistency levels 1 to 3 as acceptable—that is, plausible within the range of tolerance. As for MCR caseloads with consistency level 4, we marked them as suspicious and in need of further processing.

### Data correction process

We performed a distinct process of data correction for MCR caseloads with *availability level I* classified as *consistency level 4* and with *availability level II*. To maintain dataset completeness, we did not delete incomplete or implausible data. Instead, we corrected the values uniformly by choosing the most plausible value given in the report, rather than estimating a substitute value. If no preference could be made, the original MCR caseload was accepted as valid.

*Data availability level II* (see arrow B in Fig. 2): For the knee, if both reference variables are available, then their consistency makes the given value plausible as MCR caseload. For the other three procedures, without the possibility of cross-field comparisons within the current report, information can be taken from the relevant association report in case of hospitals with multiple sites (see arrows (iv), (v), (vi) in Fig. 1) and from the linked reports of different years of the same hospital (see arrow (vii) in Fig. 1). We used two criteria to support or reject the available (non-zero) versus zero caseload statement:

- Practicability: The only available key variable is plausible if, it is more tolerantly regulated in this study, that at least one competent medical department is available in one of the hospital sites in the association (see Table S1 for details of the departments assigned as competent). If the only available key variable is the OPS frequency and it is documented in the medical department competent for the respective MCR procedure, then the OPS frequency is more likely to be plausible than the MCR caseload of 0.
- Probability: A non-zero caseload statement in the current year is more likely to be plausible than an MCR caseload of 0, if the MCR caseload is greater than 0 in one or more other years.

*Data availability level I with consistency level 4* (see arrow C in Fig. 2): We set seven criteria for rejecting the value of a key variable. This results in the value(s) of the other available variables being confirmed or 0 being more plausible:

- If the hospital site has no profession-relevant competent medical departments (see Table S1 for details) available
- If the OPS frequency is reported identically in a competent medical department and an incompetent department (duplicate error)
- If the reported MCR caseload repeats the case number given in the previous year’s report (duplicate error, arrow (vii) in Fig. 1)
- If the reported MCR caseload repeats the case number of another hospital site in the same association (duplicate error, arrows (iv), (v), (vi) in Fig. 1)
- If the case number given for the knee QI ‘indication for elective knee endoprosthesis – primary implantation’ is incorrectly aggregated to include unicondylar replacement or revised total knee replacement
- If the case number is reported at association level rather than separately at site level (arrows (iv), (v), (vi) in Fig. 1)
- Other mistakes, for example, the value is much too high, the MCR caseload is filled in erroneously under the field of another MCR procedure

The case number hidden due to data protection was estimated as 1.5. This estimation used data pairs of OPS frequency and MCR caseload in hospital sites with data consistency level 1 for oesophagus, pancreas, and stem cells. Knee was not included in the estimation due to the presence of another key variable and the related complexity of the consistency determination.

### Data completeness

We compared the original and validated MCR caseloads to summarise the frequency of corrections done, that is, changes in caseload value and MCR relevance and compliance. The changes gave an indication of GHQR data completeness.

To analyse the error sources, we categorised both, the original and validated MCR caseloads into three groups: 0 (MCR caseload = 0), S (0 < MCR caseload < MCR threshold), and G (MCR caseload ≥ MCR threshold). Considering the categorisation before and after validation, we obtained eight combinations of changes to describe the discrepancy between original and validated data: 0–S, S–0, 0–G, G–0, S–G, G–S, S–S, G–G (see section Results). For example, 0–S: no MCR caseload reported in the original hospital site report but corrected to an MCR caseload smaller than the MCR threshold upon validation.

### Data source 2: DRG data

We compared our validated data with the DRG statistics as an external secondary data source. This allowed us to assess the outcome of our data validation.

The DRG data from the Federal Statistical Office of Germany is a dataset of all remunerated inpatient cases per year in Germany with information on diagnoses, the performed OPS, and the hospital (IIC) and hospital sites of discharge [18]. In the DRG data, hospital sites are the discharging sites, not the procedure performing sites as defined by MCR. Therefore, DRG data cannot be used for a site-level MCR-related analysis. The DRG data comply with a higher data privacy level compared with the GHQR data. The hospitals with their IIC are pseudonymised. This prevents the identification of individual hospitals and impedes the comparison of individual hospitals/hospital sites across the two datasets. Further, as mentioned in section ‘Data source 1: GHQR data’, owing to the anonymisation, hospitals with more than one IIC are counted as several hospitals. Nonetheless, the number of such hospitals is small, and this impact can be considered limited.

For the aforementioned reasons, we determined MCR compliance with DRG data based on the total OPS number in each pseudonymised IIC and, for GHQR data, based on the original and validated MCR caseload at the hospital site level as defined by MCR. We compared the GHQR and DRG caseload data of all hospitals/hospital sites at the aggregated federal level separately for each of the four MCR procedures and for each year from 2016 to 2021. The total annual federal caseload per MCR procedure was divided into two groups: cases from all hospitals/hospital sites that complied with the MCR, and cases from all hospitals/hospital sites that did not comply. We carried out all the DRG data calculations in guest research workstations at the Federal Statistical Office of Germany, with SAS Enterprise Guide 7.13 HF6.

## Results

### Detection process

The secondary dataset of GHQR consisted of 14,247 site reports with an increasing annual number from 2,275 in 2016 to 2,538 in 2021. This may be attributable to a greater degree of compliance with reporting requirements as requested by the Federal Joint Committee. Tables 1, 2, 3 and 4 provides the availability and consistency detection results. Each year, 319–421 site reports are identified as potentially relevant to the MCR-oesophagus (data availability levels I and II). Similarly, there are 548–633 site reports for the pancreas, 92–102 for stem cells, and 997–1111 for the knee. These hospital sites serve as the basis for calculating the percentage of detection and data-correction results.

**Table 1:** Statistics of internal validation results: description of data quality detection and frequency of data corrections

**Data description and evaluation**	**2016**	**2017**	**2018**	**2019**	**2020**	**2021**	**Total**
**Detection process (oesophagus**^**a**^**)**							
Number of hospital sites with GHQR report	2275	2297	2300	2299	2538	2538	14247
Number of hospital sites without MCR-relevant statements in any variables (availability level III)	1854	1906	1944	1945	2219	2199	12067
Number of hospital sites with possible relevance to MCR (availability level I + II)	421	391	356	354	319	339	2180 (100%)
Number of hospital sites with MCR caseload statements consistency level 1 (consistent)	236	234	242	245	222	190	1369 (62.8%)
Number of hospital sites with MCR caseload statements consistency level 2 (consistent)	29	32	21	21	34	37	174 (8.0%)
Number of hospital sites with MCR caseload statements consistency level 3 (consistent)	6	3	3	8	7	5	32 (1.5%)
Number of hospital sites with MCR caseload statements consistency level 4 (inconsistent)	5	4	3	0	1	9	22 (1.0%)
Number of hospital sites with only ‘MCR caseload’ given, no further statements in other relevant variables (inconsistent)	19	13	13	11	8	6	70 (3.2%)
Number of hospital sites without ‘MCR caseload’ given, but with statements in other relevant variables (inconsistent)	126	105	74	69	47	92	513 (23.5%)
**Data corrections (before and after contrast, oesophagus**^**a**^**)**							
Frequency of correction: Number of hospital sites with MCR caseload statement corrected	127	109	79	67	45	90	517 (23.7%)
MCR relevance: Number of MCR-relevant hospital sites (original, before validation)	295	286	280	283	271	247	1662
MCR relevance: Number of MCR-relevant hospital sites (validated, after validation)	404	375	343	344	303	326	2095
MCR relevance: Change in number of MCR-relevant hospital sites (contrast)	109	89	63	61	32	79	433 (19.9%)
MCR-compliant: Number of MCR-compliant hospital sites (original, before validation)	173	176	188	191	175	149	1052
MCR-compliant: Number of MCR-compliant hospital sites (validated, after validation)	176	180	185	189	175	153	1058
MCR-compliant: Change in number of MCR-compliant hospital sites (contrast)	3	4	-3	-2	0	4	6
MCR-non-compliant: Number of MCR-non-compliant hospital sites (original, before validation)	122	110	92	92	96	98	610
MCR-non-compliant: Number of MCR-non-compliant hospital sites (validated, after validation)	228	195	158	155	128	173	1037
MCR-non-compliant: Change in number of MCR-non-compliant hospital sites (contrast)	106	85	66	63	32	75	427
MCR caseload: Total MCR caseload in all hospital sites (original, before validation)	4399	4387	4753	4606	4484	4108	26737
MCR caseload: Total MCR caseload in all hospital sites (validated, after validation)	4498	4638	4793	4767	4617	4425	27738
MCR caseload: Change in total MCR caseload in all hospital sites (contrast)	99	251	40	161	133	317	1001

^a^Abbreviated from complex oesophageal interventions, complex pancreatic interventions, stem cell transplantation, and total knee replacement, respectively

**Table 2:** Statistics of internal validation results: description of data quality detection and frequency of data corrections

**Data description and evaluation**	**2016**	**2017**	**2018**	**2019**	**2020**	**2021**	**Total**
**Detection process (pancreas**^**a**^**)**							
Number of hospital sites with GHQR report	2275	2297	2300	2299	2538	2538	14247
Number of hospital sites without MCR-relevant statements in any variables (availability level III)	1642	1679	1698	1734	1990	1990	10733
Number of hospital sites with possible relevance to MCR (availability level I + II)	633	618	602	565	548	548	3514 (100%)
Number of hospital sites with MCR caseload statements consistency level 1 (consistent)	381	413	447	440	415	384	2480 (70.6%)
Number of hospital sites with MCR caseload statements consistency level 2 (consistent)	64	48	41	35	37	48	273 (7.8%)
Number of hospital sites with MCR caseload statements consistency level 3 (consistent)	17	8	10	13	10	10	68 (1.9%)
Number of hospital sites with MCR caseload statements consistency level 4 (inconsistent)	8	4	10	3	7	11	43 (1.2%)
Number of hospital sites with only ‘MCR caseload’ given, no further statements in other relevant variables (inconsistent)	13	13	16	9	10	9	70 (2.0%)
Number of hospital sites without ‘MCR caseload’ given, but with statements in other relevant variables (inconsistent)	150	132	78	65	69	86	580 (16.5%)
**Data corrections (before and after contrast, pancreas**^**a**^**)**							
Frequency of correction: Number of hospital sites with MCR caseload statement corrected	140	122	71	59	65	79	536 (15.3%)
MCR relevance: Number of MCR-relevant hospital sites (original, before validation)	483	486	523	498	479	462	2931
MCR relevance: Number of MCR-relevant hospital sites (validated, after validation)	609	592	573	550	534	531	3389
MCR relevance: Change in number of MCR-relevant hospital sites (contrast)	126	106	50	52	55	69	458 (13.0%)
MCR-compliant: Number of MCR-compliant hospital sites (original, before validation)	389	388	393	381	370	363	2284
MCR-compliant: Number of MCR-compliant hospital sites (validated, after validation)	401	395	390	379	367	363	2295
MCR-compliant: Change in number of MCR-compliant hospital sites (contrast)	12	7	-3	-2	-3	0	11
MCR-non-compliant: Number of MCR-non-compliant hospital sites (original, before validation)	94	98	130	117	109	99	647
MCR-non-compliant: Number of MCR-non-compliant hospital sites (validated, after validation)	208	197	183	171	167	168	1094
MCR-non-compliant: Change in number of MCR-non-compliant hospital sites (contrast)	114	99	53	54	58	69	447
MCR caseload: Total MCR caseload in all hospital sites (original, before validation)	11547	11685	12119	12237	12379	12196	72163
MCR caseload: Total MCR caseload in all hospital sites (validated, after validation)	12100	12221	12233	12301	12349	12418	73622
MCR caseload: Change in total MCR caseload in all hospital sites (contrast)	553	536	114	64	-30	222	1459

^a^Abbreviated from complex oesophageal interventions, complex pancreatic interventions, stem cell transplantation, and total knee replacement, respectively

**Table 3:** Statistics of internal validation results: description of data quality detection and frequency of data corrections

**Data description and evaluation**	**2016**	**2017**	**2018**	**2019**	**2020**	**2021**	**Total**
**Detection process (stem cells**^**a**^**)**							
Number of hospital sites with GHQR report	2275	2297	2300	2299	2538	2538	14247
Number of hospital sites without MCR-relevant statements in any variables (availability level III)	2173	2196	2204	2207	2440	2440	13660
Number of hospital sites with possible relevance to MCR (availability level I + II)	102	101	96	92	98	98	587 (100%)
Number of hospital sites with MCR caseload statements consistency level 1 (consistent)	56	63	64	59	59	68	369 (62.9%)
Number of hospital sites with MCR caseload statements consistency level 2 (consistent)	23	21	20	21	21	16	122 (20.8%)
Number of hospital sites with MCR caseload statements consistency level 3 (consistent)	5	1	5	4	6	1	22 (3.7%)
Number of hospital sites with MCR caseload statements consistency level 4 (inconsistent)	2	1	1	0	0	2	6 (1.0%)
Number of hospital sites with only ‘MCR caseload’ given, no further statements in other relevant variables (inconsistent)	2	5	3	4	3	3	20 (3.4%)
Number of hospital sites without ‘MCR caseload’ given, but with statements in other relevant variables (inconsistent)	14	10	3	4	9	8	48 (8.2%)
**Data corrections (before and after contrast, stem cells**^**a**^**)**							
Frequency of correction: Number of hospital sites with MCR caseload statement corrected	9	12	6	4	10	7	48 (8.2%)
MCR relevance: Number of MCR-relevant hospital sites (original, before validation)	88	91	93	88	89	90	539
MCR relevance: Number of MCR-relevant hospital sites (validated, after validation)	95	94	92	88	89	89	547
MCR relevance: Change in number of MCR-relevant hospital sites (contrast)	7	3	-1	0	0	-1	8 (1.4%)
MCR-compliant: Number of MCR-compliant hospital sites (original, before validation)	67	70	74	76	81	83	451
MCR-compliant: Number of MCR-compliant hospital sites (validated, after validation)	68	71	73	76	77	80	445
MCR-compliant: Change in number of MCR-compliant hospital sites (contrast)	1	1	-1	0	-4	-3	-6
MCR-non-compliant: Number of MCR-non-compliant hospital sites (original, before validation)	21	21	19	12	8	7	88
MCR-non-compliant: Number of MCR-non-compliant hospital sites (validated, after validation)	27	23	19	12	12	9	102
MCR-non-compliant: Change in number of MCR-non-compliant hospital sites (contrast)	6	2	0	0	4	2	14
MCR caseload: Total MCR caseload in all hospital sites (original, before validation)	7308	7776	8267	8333	8405	8164	48253
MCR caseload: Total MCR caseload in all hospital sites (validated, after validation)	7766	7913	8197	8296	8108	8324	48604
MCR caseload: Change in total MCR caseload in all hospital sites (contrast)	458	137	-70	-37	-297	160	351

^a^Abbreviated from complex oesophageal interventions, complex pancreatic interventions, stem cell transplantation, and total knee replacement, respectively

**Table 4:** Statistics of internal validation results: description of data quality detection and frequency of data corrections

**Data description and evaluation**	**2016**	**2017**	**2018**	**2019**	**2020**	**2021**	**Total**
**Detection process (knee**^**a**^**)**							
Number of hospital sites with GHQR report	2275	2297	2300	2299	2538	2538	14247
Number of hospital sites without MCR-relevant statements in any variables (availability level III)	1164	1214	1250	1267	1523	1541	7959
Number of hospital sites with possible relevance to MCR (availability level I + II)	1111	1083	1050	1032	1015	997	6288 (100%)
Number of hospital sites with MCR caseload statements consistency level 1 (consistent)	548	708	774	776	740	733	4279 (68.1%)
Number of hospital sites with MCR caseload statements consistency level 2 (consistent)	171	140	107	137	133	137	825 (13.1%)
Number of hospital sites with MCR caseload statements consistency level 3 (consistent)	140	60	71	53	62	57	443 (7.0%)
Number of hospital sites with MCR caseload statements consistency level 4 (inconsistent)	62	43	39	20	24	21	209 (3.3%)
Number of hospital sites with only ‘MCR caseload’ given, no further statements in other relevant variables (inconsistent)	5	6	5	5	6	3	30 (0.5%)
Number of hospital sites without ‘MCR caseload’ given, but with statements in other relevant variables (inconsistent)	185	126	54	41	50	46	502 (8.0%)
**Data corrections (before and after contrast, knee**^**a**^**)**							
Frequency of correction: Number of hospital sites with MCR caseload statement corrected	180	126	58	37	49	46	496 (7.9%)
MCR relevance: Number of MCR-relevant hospital sites (original, before validation)	926	957	996	991	965	951	5786
MCR relevance: Number of MCR-relevant hospital sites (validated, after validation)	1032	1025	1009	1001	981	973	6021
MCR relevance: Change in number of MCR-relevant hospital sites (contrast)	106	68	13	10	16	22	235 (3.7%)
MCR-compliant: Number of MCR-compliant hospital sites (original, before validation)	854	881	923	905	804	794	5161
MCR-compliant: Number of MCR-compliant hospital sites (validated, after validation)	930	924	925	905	806	804	5294
MCR-compliant: Change in number of MCR-compliant hospital sites (contrast)	76	43	2	0	2	10	133
MCR-non-compliant: Number of MCR-non-compliant hospital sites (original, before validation)	72	76	73	86	161	157	625
MCR-non-compliant: Number of MCR-non-compliant hospital sites (validated, after validation)	102	101	84	96	175	169	727
MCR-non-compliant: Change in number of MCR-non-compliant hospital sites (contrast)	30	25	11	10	14	12	102
MCR caseload: Total MCR caseload in all hospital sites (original, before validation)	144102	145471	152188	151374	134268	131558	858961
MCR caseload: Total MCR caseload in all hospital sites (validated, after validation)	150677	150098	149860	149889	133502	132447	866473
MCR caseload: Change in total MCR caseload in all hospital sites (contrast)	6575	4627	-2328	-1485	-766	889	7512

^a^Abbreviated from complex oesophageal interventions, complex pancreatic interventions, stem cell transplantation, and total knee replacement, respectively

Most sites (72.3%) reported valid oesophagus caseloads (consistency levels 1, 2, and 3), while 27.7% reported suspicious data requiring correction (consistency level 4 and either no or only MCR caseload reported). The corresponding percentages of sites entering the correction process are 19.7%, 12.6%, and 11.8% for the pancreas, stem cells, and knee, respectively (see the ‘Detection process’ panel in Table 1, marked ‘inconsistent’).

### Data correction process

We processed the suspicious values using the plausibility criteria described in section ‘Data correction process’ and Fig. 2. The results are also provided in Table 1. A total of 517 hospital sites (23.7%) have had their oesophagus caseloads corrected. For the pancreas, stem cells, and knee, the total numbers of corrections are 536 (15.3%), 48 (8.2%), and 496 (7.9%), respectively.

### Data completeness

The contrast between the original and corrected caseloads are listed in the ‘Data corrections’ panel in Table 1. Compared with the originally reported caseloads, the total corrected caseloads over the six years increased for the four MCR procedures (Table 1 ‘MCR caseload’), and the number of MCR-relevant hospital sites increased as well (Table 1 ‘MCR relevance’). Therefore, the completeness of the total caseload was 96.3%, 98.0%, 99.3%, and 99.1% for the oesophagus, pancreas, stem cells, and knee, respectively, whereas the completeness of relevant hospital sites was 73.9%, 84.4%, 98.5%, and 95.9%, respectively.

### Error sources

Based on the categorisation of original and validated MCR caseloads, we noted the following findings on the error sources (see Table 5):

**Table 5:** Contrast of MCR caseload correction, categorised in eight groups

		**2016**	**2017**	**2018**	**2019**	**2020**	**2021**
**0–S: No report on MCR-non-compliant caseload**							
**Oesophagus**	Hospital^a^	110	88	69	63	35	77
	Case^b^	333	249	187	196	104	228
**Pancreas**	Hospital^a^	114	99	57	55	59	68
	Case^b^	346	296	151	141	148	184
**Stem cells**	Hospital^a^	6	5	2	2	4	2
	Case^b^	18	17	7	9	8	21
**Knee**	Hospital^a^	28	27	12	10	15	13
	Case^b^	385	296	83	128	103	95
**S–0: MCR-non-compliant caseload reported, but not valid**							
**Oesophagus**	Hospital^a^	6	4	3	0	3	0
	Case^b^	-28	-13	-22	0	-15	0
**Pancreas**	Hospital^a^	1	1	3	0	1	0
	Case^b^	-1	-2	-18	0	-1	0
**Stem cells**	Hospital^a^	0	4	3	2	0	0
	Case^b^	0	-46	-59	-46	0	0
**Knee**	Hospital^a^	3	4	0	2	3	2
	Case^b^	-14	-7	0	-43	-100	-96
**0–G: No report on MCR-compliant caseload**							
**Oesophagus**	Hospital^a^	7	9	1	1	3	5
	Case^b^	249	203	10	11	44	143
**Pancreas**	Hospital^a^	18	14	1	0	0	4
	Case^b^	582	357	12	0	0	227
**Stem cells**	Hospital^a^	2	2	0	0	1	1
	Case^b^	469	184	0	0	35	415
**Knee**	Hospital^a^	88	51	5	4	7	12
	Case^b^	12798	8058	384	352	951	1917
**G–0: MCR-compliant caseload reported, but not valid**							
**Oesophagus**	Hospital^a^	2	4	4	3	3	3
	Case^b^	-180	-90	-65	-46	-70	-94
**Pancreas**	Hospital^a^	5	6	5	3	3	3
	Case^b^	-99	-116	-89	-89	-173	-185
**Stem cells**	Hospital^a^	1	0	0	0	5	4
	Case^b^	-29	0	0	0	-340	-276
**Knee**	Hospital^a^	7	6	4	2	3	1
	Case^b^	-1506	-1041	-688	-492	-564	-333
**S–G: MCR-compliant caseload reported as non-compliant**							
**Oesophagus**	Hospital^a^	0	0	0	0	0	2
	Case^b^	0	0	0	0	0	21
**Pancreas**	Hospital^a^	0	0	2	1	0	0
	Case^b^	0	0	16	12	0	0
**Stem cells**	Hospital^a^	0	0	0	0	0	0
	Case^b^	0	0	0	0	0	0
**Knee**	Hospital^a^	0	1	1	0	1	0
	Case^b^	0	195	25	0	22	0
**G–S: MCR-non-compliant caseload reported as compliant**							
**Oesophagus**	Hospital^a^	2	1	0	0	0	0
	Case^b^	-275	-9	0	0	0	0
**Pancreas**	Hospital^a^	1	1	1	0	0	1
	Case^b^	-16	-10	-17	0	0	-8
**Stem cells**	Hospital^a^	0	1	1	0	0	0
	Case^b^	0	-18	-18	0	0	0
**Knee**	Hospital^a^	5	3	0	2	3	1
	Case^b^	-270	-452	0	-49	-206	-22
**S–S: False MCR caseload reported (before and after: both MCR-non-compliant)**							
**Oesophagus**	Hospital^a^	0	1	0	0	0	0
	Case^b^	0	3	0	0	0	0
**Pancreas**	Hospital^a^	0	0	0	0	0	0
	Case^b^	0	0	0	0	0	0
**Stem cells**	Hospital^a^	0	0	0	0	0	0
	Case^b^	0	0	0	0	0	0
**Knee**	Hospital^a^	1	3	0	1	2	2
	Case^b^	15	-28	0	-6	0	-36
**G–G: False MCR caseload reported (before and after: both MCR-compliant)**							
**Oesophagus**	Hospital^a^	0	2	2	0	1	3
	Case^b^	0	-92	-70	0	70	19
**Pancreas**	Hospital^a^	1	1	2	0	2	3
	Case^b^	-259	11	59	0	-4	4
**Stem cells**	Hospital^a^	0	0	0	0	0	0
	Case^b^	0	0	0	0	0	0
**Knee**	Hospital^a^	48	31	36	16	15	15
	Case^b^	-4833	-2394	-2132	-1375	-972	-636

^a^Number of hospital sites with corrected caseload values

^b^Sum of change in caseloads

1. The most frequent corrections occurred for the combination of 0–S. The cases performed, whose total number had not reached the MCR threshold, were not reported as caseloads in the MCR section, only in the OPS or knee QI section of the GHQR.
2. Some hospitals did not report their MCR caseloads, even when they had reached MCR thresholds (0–G). This was observed in 2016 and 2017, when plausibility control rules had not been introduced in the MCR section of the GHQR.
3. In some cases, although their MCR caseloads reached MCR thresholds both before and after data correction (G–G), hospitals documented a wrong case number. We identified the reasons as follows: first, misunderstanding of the MCR caseload delimitation (hospital site, not association); second, use of the incorrect OPS list (e.g. incorrect inclusion of unicondylar and revised total knee replacement, resulting in incorrect case numbers for each MCR procedure); and lastly, erroneously taking over caseloads from another site in the same association or from the past year’s report.
4. Regarding G–0, the reasons for the mistakes were caseloads being taken over from another site in the same association (stem cells and knee) or mistakenly taken from the past year report (knee). Especially for stem cells, two hospital sites, which only treated patients under 18 years old, entered their case numbers incorrectly into their MCR caseload. According to the MCR for oesophagus and stem cells, hospital sites that treat only children are not restricted by the MCR.
5. In some cases, the caseload difference between pre- and post-validation was large because the hospital forgot to report a caseload of 300 or mistakenly entered 30 as 300. Only a few hospitals made such mistakes, but they made a relatively large discrepancy in the total caseloads. Some other errors with no visible trace were considered as unintentional/random.

### External comparison with DRG data

**Fig. 3:**
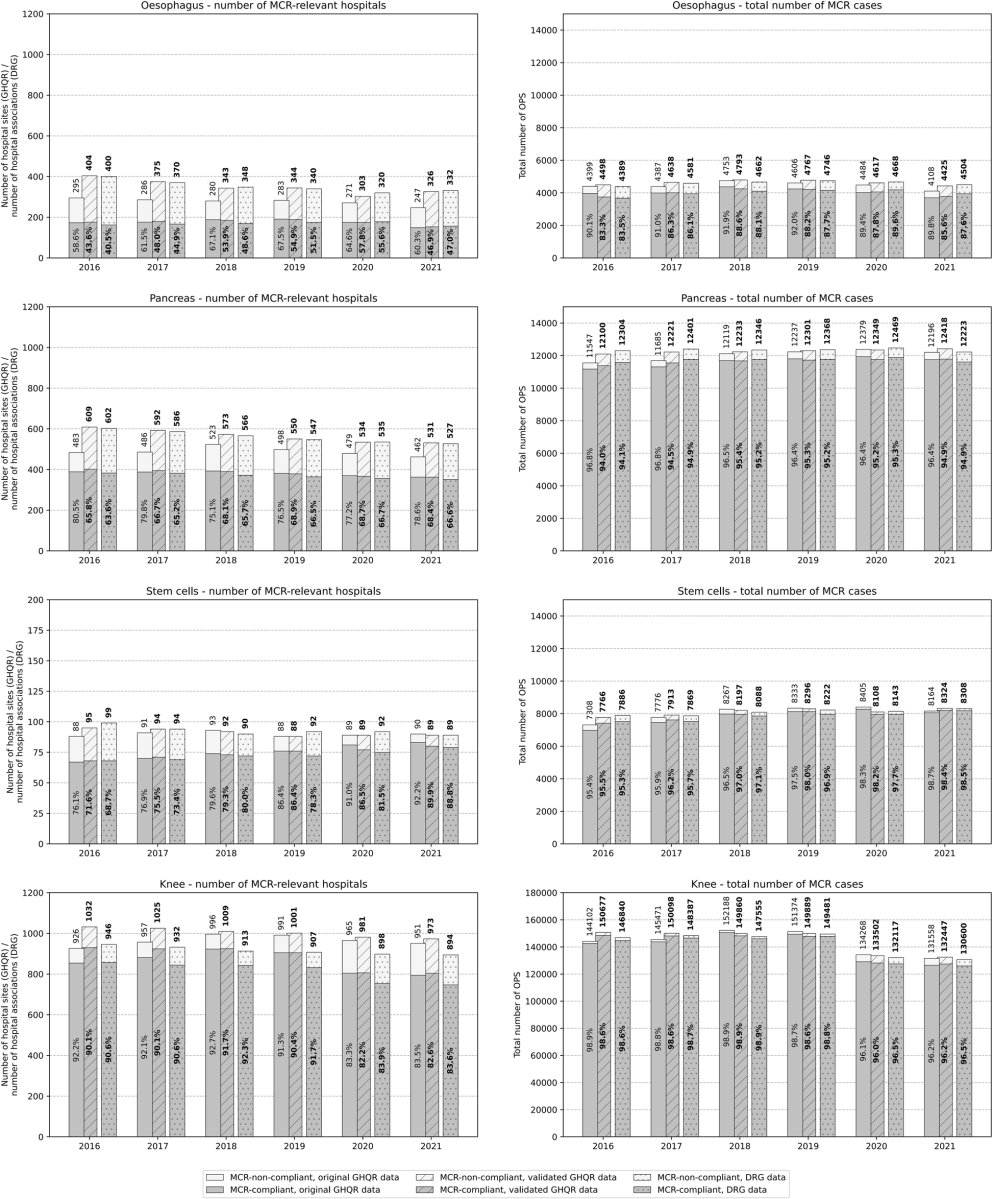
Hospital compliance with minimum caseload requirements (MCR) in Germany for the four procedures for the period 2016–2021, before and after GHQR data validation, compared with Diagnosis-Related Groups Statistic (DRG) data from the Federal Statistical Office of Germany. Left figure section: number of hospitals per year, right figure section: number of MCR cases in Germany per year; in all figures: left and middle columns GHQR data on hospital site level, right columns DRG data on IIC level)

Figure 3 illustrates the magnitude of change before and after validation. Hospitals’ MCR compliance in DRG data was determined at the IIC level (≈ hospital association level), whereas in the GHQR data, it was determined at the hospital site level. However, for the four MCR procedures, 98.5% (oesophagus), 93.8% (pancreas), 95.4% (stem cells) and 82.9% (knee) of hospital sites in the GHQR correspond directly to the IIC level in the DRG data, because there is only one performing site at the IIC level. Nonetheless, the counting of hospital sites in the GHQR results is systematically greater compared to the number of hospital associations at IIC level in the DRG data. 1.5% (oesophagus), 6.2% (pancreas), 4.6% (stem cells), and 17.1% (knee) of hospital sites are located in hospital associations with more than one performing site.

Our validation resulted in a systematically higher number of hospital sites being included in the validated data compared to the original data. This outcome aligns with the number of hospitals in the DRG dataset (Fig. 3, left). The number of hospital sites in the validated data for oesophagus, pancreas, and stem cells is equal to or slightly higher than the number of hospitals in the DRG data defined by the hospital association IIC level, which is almost identical for these procedures, as previously stated. For knee, the validation also resulted in a higher number of hospital sites, but the comparison to the DRG hospital numbers shows about 8.1% to 9.5% fewer hospitals at the IIC level as defined in the DRG data. This corresponds to the aforementioned higher percentage of hospital associations in knee procedures with more than one performing hospital site.

Further, most of the hospital sites identified through the validation process perform caseloads below the MCR thresholds (Fig. 3, left). This confirms the error source analysis.

On a federal level, the case numbers of the DRG data confirm the validated DRG data within a range of 1% (Fig. 3, right).

As supplementary information, the validated GHQR data were also aggregated at the IIC level, which is not the site level required by MCR policy but allows for comparison at the same aggregation level as DRG data (see Table S2 in the Supplementary Material).

## Discussion

### Discussion of results

This study quantitatively assessed the data validity of MCR caseloads in the secondary data source GHQR, confirming the data incompleteness due to underreporting of hospitals with small MCR caseloads below the MCR thresholds, as stated in earlier studies [14–17].

Our validation substantially corrected the MCR values of the original GHQR data. We adjusted the caseloads of 7.9%–23.7% hospital sites for the four MCR procedures under study. In turn, 1.4%–19.9% of hospital sites and 0.7%–3.7% of total caseloads were added to the GHQR MCR caseloads. These results of our internal validation process could be confirmed via comparison with the external DRG data.

Based on our findings, we recommend that researchers undertake a validation process before using MCR caseload data in the GHQR, especially for GHQR data before 2018 that lacks plausibility control by the Federal Joint Committee. Based on our error source analysis, we suggest an adapted plausibility control to improve the quality of MCR-related documentation by comparing the number of MCR-relevant OPS with the MCR caseload. This plausibility rule could work with a certain tolerance range and could help to avoid typical errors such as no reporting of non-compliance, reporting caseloads at the association level rather than at the hospital site level, and some random errors.

Our study period included hospital data from 2020 and 2021, two years significantly impacted by the COVID-19 pandemic, which affected patients and their treatments in hospitals. Concerning hospital documentation, the Federal Joint Committee specified in its ‘Note on the COVID-19 pandemic in the reporting years 2020 and 2021’ that hospitals were required to report caseloads in the GHQR and document when the pandemic prevented meeting the MCR, coding these as exceptional circumstances [13]. Based on this requirement, we suggest that the impact of the COVID-19 pandemic on the validity of GHQR data was minimal.

### Discussion of methods

Due to the importance of data completeness for health services research and especially for the topic of MCR, this study employed a comprehensive approach that considered all administrative levels, time periods and logical plausibility to facilitate the data validation process. This method ensured data completeness by selecting the most plausible values without discarding incomplete or implausible data. Specifically, we carried out the internal cross-field validation of MCR caseloads reported in GHQR 2016–2021 on five dimensions: across different variables (1) at the site level within a site report, (2) between the site and department levels, (3) between the hospital association and site levels, (4) with a time dimension spanning the six years from 2016 to 2021, and (5) with the general logical and medical practicality. The first two dimensions were used for the detection process, and the last three were used for the data corrections.

We detected the mathematical inconsistencies and implausibilities and set a tolerance range to tolerate possible practical deviations due to the counting method, documentation, and some other sources of inaccuracy. In the tolerance range, the slightly different values of the key variables cannot be distinguished by plausibility. In this situation, we kept the original values. We corrected the MCR caseloads only when the values of the key variables were outside the tolerance range, and there was sufficient evidence from the cross-field comparison indicating that the corrected values would be more plausible than the original values. We sought to preserve data as original as possible and keep the influence on the GHQR data as little as possible.

The method used to assess validation outcomes in this study is tailored to the research focus on MCR. Validated caseloads are categorised into groups of compliant and non-compliant hospitals. This assessment can be structured differently to accommodate other research interests.

This research integrated multiple methodological elements for data validation, including comparison and correction principles and processes, multi-dimensional analysis, and tolerance ranges, each of which can be applied independently. These elements are generalisable to research involving diverse datasets or topics, provided the dataset possesses ‘rich and multifaceted characteristics’. This prerequisite implies that, if data richness and multifacetedness are maintained across datasets, this approach may also be extended to cross-dataset data validation—external validation.

Guidelines for reporting secondary data analyses [1, 2] call for a statement on data validity to address potential biases and limitations, but they do not specifically require reporting on the validation process used. We encourage researchers who choose to use a validation process in their secondary data analyses to describe all steps of their detection process, as well as any correction process applied. This transparency allows readers to understand whether data validity is merely described as a limitation, or a specific validation process has been applied.

### Limitations

The accuracy of the MCR caseload validation is restricted for two reasons: (1) the data protection of small OPS case numbers and (2) the (not full) comparability of the counting method of MCR caseloads defined by OPS with the mere OPS frequency. The value inconsistencies based on these two reasons are not errors but, rather, are due to the nature of this kind of data. However, together with unintentional/random errors made by hospitals in reporting data, the small discrepancies between the variables are difficult to attribute to data nature or error.

In general, internal validation is limited by the lack of cross-source data comparison. As such, the validation outcome depends highly on the submission and plausibility control of the data source. In this study, we could compare the outcome of the internally validated MCR caseloads from the GHQR with the DRG data as an external data source. However, due to the IIC pseudonymisation of DRG data, the external comparison involves two different levels for categorising and counting of hospitals and case numbers: hospital association level for DRG data and site level for GHQR data. Consequently, the confirmation of the internal validation outcome is limited to the aggregated level.

## Conclusions

Our study established an internal validation process for the caseloads of four MCR procedures reported in the GHQR. The validation process design was based on the dataset’s rich and multifaceted characteristics. The validation considered the Good Practice of Secondary Data Analysis as guidelines and used cross-field comparison as the main approach. Using this validation process, we evaluated dataset completeness and analysed the error sources of the MCR case numbers in GHQR. This internal validation process could be adapted and applied to research questions analysing different variables within the GHQR or other secondary data sources, provided there is sufficient redundant information available in the dataset.

## Supplementary Information

Supplementary Material 1.

## Data Availability

The German Hospital Quality Reports data for this study are available from the Federal Joint Committee upon reasonable request. Processed data will be shared on request with the corresponding author with permission from the Federal Joint Committee. The Diagnosis-Related Groups (DRG) Statistic used in this study consists of pseudonymised microdata, available via remote execution or on-site use in guest research workstations.

## Abbreviations

GHQR: German Hospital Quality Reports
MCR: Minimum caseload requirements
OPS: Operation and Procedure Classification Codes (OPS codes, from the German term Operationen- und Prozedurenschlüssel)
DRG: Diagnosis-Related Groups Statistic
ICD: International Classification of Diseases
QI: Quality indicators in GHQR
IIC: Institution identification code
