# Supplementary material for: Internal validation of self-reported case numbers in hospital quality reports: preparing secondary data for health services research
Source: BMC Med Res Methodol. 2024 Dec 31;24:325. doi: 10.1186/s12874-024-02429-6 (PMC11686984; doi:10.1186/s12874-024-02429-6)
Supplement: Supplementary file 1 — Supplementary Material 1. [file 12874_2024_2429_MOESM1_ESM.docx]

**Table S1**: Medical departments assigned as competent for each MCR procedure and the corresponding medical department code

|  | **Oesophagus** | **Pancreas** | **Stem cells** | **Knee** | **Medical department code** |
| --- | --- | --- | --- | --- | --- |
| **Internal medicine** |  |  | ✓ |  | Starts with 01 |
| **Haemato-oncology** |  |  | ✓ |  | Starts or ends with 05 |
| **Surgery** | ✓ | ✓ |  | ✓ | Starts with 15 |
| **Accident surgery** | ✓ | ✓ |  | ✓ | Starts with 16 |
| **Thoracic surgery** | ✓ | ✓ |  |  | Starts with 20 |
| **Cardiac surgery** | ✓ |  |  |  | Starts with 21 |
| **Orthopaedics** |  |  |  | ✓ | Starts with 23 |
| **Visceral surgery** | ✓ | ✓ |  |  | 3757 |

**Table S2:** Validated case numbers and number of MCR-related hospitals aggregated at the IIC level

|  | **Year** | **Number of IIC** | | | | | | **Number of cases** | | | | |
| --- | --- | --- | --- | --- | --- | --- | --- | --- | --- | --- | --- | --- |
|  |  | **with more than one site** | **MCR-compliant** | | **MCR-non-compliant** | | **total** | **MCR-compliant** | | **MCR-non-compliant** | | **total** |
| Oesophagus | 2016 | 7 | 178 | (45.2%) | 216 | (54.8%) | 394 | 3798 | (84.4%) | 700 | (15.6%) | 4498 |
|  | 2017 | 8 | 179 | (49.2%) | 185 | (50.8%) | 364 | 4026 | (86.8%) | 612 | (13.2%) | 4638 |
|  | 2018 | 7 | 179 | (53.6%) | 155 | (46.4%) | 334 | 4249 | (88.7%) | 544 | (11.3%) | 4793 |
|  | 2019 | 8 | 187 | (56.0%) | 147 | (44.0%) | 334 | 4241 | (89.0%) | 526 | (11.0%) | 4767 |
|  | 2020 | 6 | 173 | (58.2%) | 124 | (41.8%) | 297 | 4075 | (88.3%) | 542 | (11.7%) | 4617 |
|  | 2021 | 6 | 152 | (47.6%) | 167 | (52.4%) | 319 | 3802 | (85.9%) | 623 | (14.1%) | 4425 |
| Pancreas | 2016 | 16 | 397 | (67.7%) | 189 | (32.3%) | 586 | 11439 | (94.5%) | 661 | (5.5%) | 12100 |
|  | 2017 | 9 | 387 | (67.1%) | 190 | (32.9%) | 577 | 11571 | (94.7%) | 650 | (5.3%) | 12221 |
|  | 2018 | 19 | 381 | (69.4%) | 168 | (30.6%) | 549 | 11712 | (95.7%) | 521 | (4.3%) | 12233 |
|  | 2019 | 10 | 372 | (69.7%) | 162 | (30.3%) | 534 | 11755 | (95.6%) | 546 | (4.4%) | 12301 |
|  | 2020 | 15 | 359 | (69.7%) | 156 | (30.3%) | 515 | 11771 | (95.3%) | 578 | (4.7%) | 12349 |
|  | 2021 | 14 | 358 | (69.9%) | 154 | (30.1%) | 512 | 11827 | (95.2%) | 591 | (4.8%) | 12418 |
| Stem cells | 2016 | 0 | 86 | (90.5%) | 9 | (9.5%) | 95 | 7736 | (99.6%) | 30 | (0.4%) | 7766 |
|  | 2017 | 0 | 86 | (91.5%) | 8 | (8.5%) | 94 | 7882 | (99.6%) | 31 | (0.4%) | 7913 |
|  | 2018 | 1 | 84 | (92.3%) | 7 | (7.7%) | 91 | 8165 | (99.6%) | 32 | (0.4%) | 8197 |
|  | 2019 | 1 | 83 | (95.4%) | 4 | (4.6%) | 87 | 8285 | (99.9%) | 11 | (0.1%) | 8296 |
|  | 2020 | 1 | 83 | (94.3%) | 5 | (5.7%) | 88 | 8095 | (99.8%) | 13 | (0.2%) | 8108 |
|  | 2021 | 1 | 87 | (98.9%) | 1 | (1.1%) | 88 | 8322 | (100.0%) | 2 | (0.0%) | 8324 |
| Knee | 2016 | 76 | 913 | (96.9%) | 29 | (3.1%) | 942 | 150580 | (99.9%) | 97 | (0.1%) | 150677 |
|  | 2017 | 82 | 900 | (97.0%) | 28 | (3.0%) | 928 | 150001 | (99.9%) | 97 | (0.1%) | 150098 |
|  | 2018 | 82 | 884 | (96.9%) | 28 | (3.1%) | 912 | 149734 | (99.9%) | 126 | (0.1%) | 149860 |
|  | 2019 | 83 | 882 | (97.8%) | 20 | (2.2%) | 902 | 149833 | (100.0%) | 56 | (0.0%) | 149889 |
|  | 2020 | 70 | 876 | (97.8%) | 20 | (2.2%) | 896 | 133438 | (100.0%) | 64 | (0.0%) | 133502 |
|  | 2021 | 67 | 874 | (97.9%) | 19 | (2.1%) | 893 | 132380 | (99.9%) | 67 | (0.1%) | 132447 |
